# Machine Learning and Quantum Calculation for Predicting Yield in Cu-Catalyzed P–H Reactions

**DOI:** 10.3390/molecules28165995

**Published:** 2023-08-10

**Authors:** Youfu Ma, Xianwei Zhang, Lin Zhu, Xiaowei Feng, Jamal A. H. Kowah, Jun Jiang, Lisheng Wang, Lihe Jiang, Xu Liu

**Affiliations:** 1 Medical College, Guangxi University, Nanning 530004, China; muduo_youfu@163.com (Y.M.); zl17851182520@163.com (L.Z.); fengxiaowei77@outlook.com (X.F.); jamal.kowah@gmail.com (J.A.H.K.); jiangjun@gxu.edu.cn (J.J.); 2 School of Basic Medical Sciences, Youjiang Medical University for Nationalities, Baise 533000, China

**Keywords:** machine learning, quantum chemistry, SVM, transition state, copper catalysts

## Abstract

The paper discussed the use of machine learning (ML) and quantum chemistry calculations to predict the transition state and yield of copper-catalyzed P–H insertion reactions. By analyzing a dataset of 120 experimental data points, the transition state was determined using density functional theory (DFT). ML algorithms were then applied to analyze 16 descriptors derived from the quantum chemical transition state to predict the product yield. Among the algorithms studied, the Support Vector Machine (SVM) achieved the highest prediction accuracy of 97%, with over 80% correlation in Leave-One-Out Cross-Validation (LOOCV). Sensitivity analysis was performed on each descriptor, and a comprehensive investigation of the reaction mechanism was conducted to better understand the transition state characteristics. Finally, the ML model was used to predict reaction plans for experimental design, demonstrating strong predictive performance in subsequent experimental validation.

## 1. Introduction

Catalysts play an essential role in various chemical transformations. However, the search for highly efficient catalysts for specific reactions remains a challenging task due to the complexity of catalytic processes [1,2,3]. One effective strategy for constructing C–C and C–heteroatom bonds is the insertion reaction of α-azido carbonyl compounds catalyzed by transition metals [4,5,6,7,8]. Another significant method for synthesizing organic phosphine compounds is the P–H insertion reaction [9]. However, there is relatively little research on P–H insertion reactions in comparison to other X–H insertion reactions, and the range of applicable metal catalysts is limited. The weak nucleophilic ability of phosphorus, along with its high susceptibility to coordination bonding with metals due to the presence of lone pair electrons in its outermost shell, poses challenges in the formation of metal carbene intermediates and the occurrence of P–H insertion reactions. Copper has emerged as a crucial catalyst in facilitating P–H insertion reactions [10,11,12,13]. Nonetheless, the complexity of the reaction, involving numerous catalysts and substrates, requires scientists to rely on their expertise and intuition, conducting trial and error experiments to identify suitable reaction conditions. Despite significant efforts and resources invested, the outcomes often prove unsatisfactory. 

In the field of catalyst design and optimization, computational chemistry has become increasingly important [14]. One strategy involves using quantum chemical methods to simulate reaction transition states [15,16,17,18,19,20,21,22,23]. However, the vast space of catalytic materials and the diversity of reaction conditions make traditional quantum mechanical-based computational chemistry inefficient for catalyst screening [24,25,26]. Fortunately, artificial intelligence (AI) technology based on machine learning algorithms can overcome these barriers, significantly accelerating the catalyst design process [27,28,29]. Integrating quantum chemistry transition-state models with machine learning in catalyst design workflows can provide valuable information, including experimental yield predictions and transition-state characteristics that may not be easily obtained through other means. 

While there has been growing interest in using machine learning and quantum chemistry calculations to parameterize experimental data and predict optimal catalytic conditions [29,30,31,32,33,34], examples of using machine learning to analyze experimental data and predict results under new reaction conditions are still limited. The combination of these two approaches to predict product yields in copper-catalyzed P–H insertion reactions is a novel application. 

**Figure 1:**
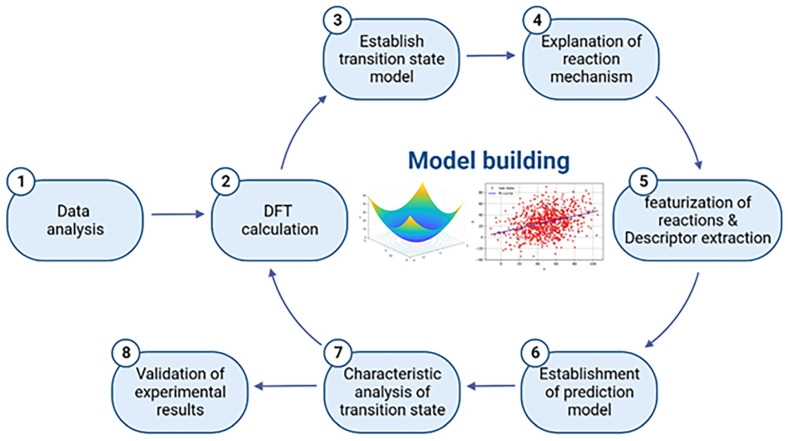
Flowchart of the machine learning methodology employed in this study.

In this study, we employ quantum chemical calculations to elucidate the transition state of the copper-catalyzed P–H insertion reaction (Figure 1). Subsequently, we integrate the quantum chemical transition state model with machine learning to predict the final outcomes of the reaction. Through quantum chemical calculations of the reaction mechanism and sensitivity analysis of important descriptors, we identify the transition state features of this reaction, which can aid future in-depth investigations. Our optimal machine learning model, the Support Vector Machine (SVM) [35,36], exhibits the highest predictive accuracy and demonstrates excellent precision and performance through subsequent experimental validation. This approach provides accurate guidance for scientists in designing and selecting optimal reaction conditions and holds promise for identifying other optimal reaction catalysts. 

## 2. Results and Discussion

A total of 110 experimental data on copper-catalyzed P–H insertion reactions were initially obtained from relevant literature. However, before extracting descriptors that accurately summarize catalyst performance, it is necessary to utilize density functional theory (DFT) to calculate and determine the transition state and reaction mechanism. Specifically, in the X–H insertion reaction of *α*-imino copper carbenes, it is important to understand why the reaction pathway is more inclined towards the 1,3-insertion pathway rather than the 1,1-insertion pathway. The literature extensively reports the DFT study of the reaction process, where diazo compounds are converted to metal carbenes under a metal catalyst [37,38,39,40,41]. 

**Figure 2:**
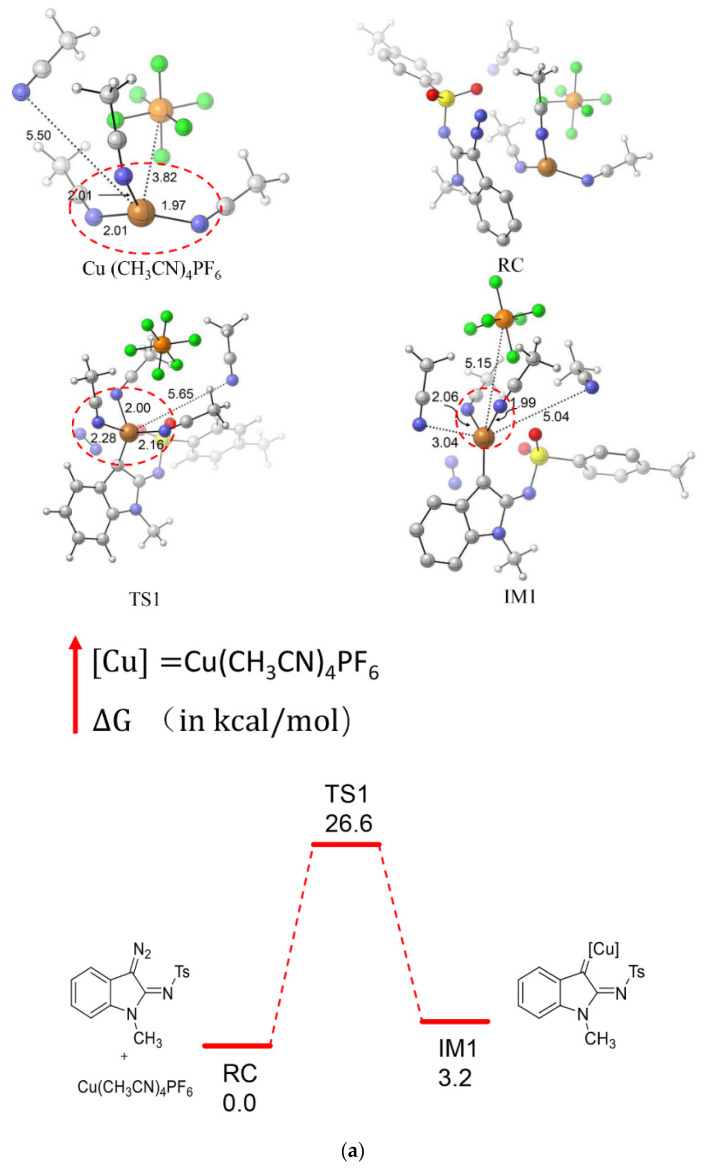

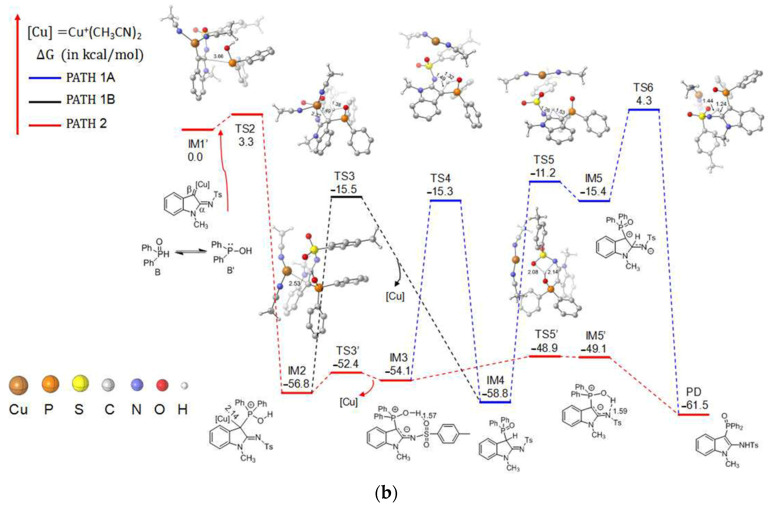
(**a**) (1) Gibbs free energy (in kcal/mol) profile obtained at the SMD_CHCl3_/M06/6–311++G(d,p)-LANL2DZ//B3LYP-D3/6-31G(d)-LANL2DZ level of theory for the formation of the α-imino copper carbene from 3-diazoindolin-2-imine catalyzed by Cu(CH_3_CN)_4_PF_6_. (2) Distance was given in Å. (3) Temperature of 323.15 K and a pressure of 1 atm. (**b**) (1) Gibbs free energy (in kcal/mol) profile obtained at the SMD_CHCl3_/M06/6–311++G(d,p)-LANL2DZ//B3LYP-D3/6-31G(d)-LANL2DZ level of theory for the formation of the 3-phosphonylindole product from α-imino copper carbene and *H*–phosphine oxides. The pathways shown in black and blue were 1,1-insertion reactions followed by hydrogen transfer processes, and the red pathway was a 1,3-insertion pathway. (2) Important intermediates and transition states considered in the computation of the energetic span for the catalytic cycle. (3) Distances were given in Å. (4) Temperature of 323.15 K and a pressure of 1 atm.

In this study, we utilized our newly synthesized catalyst, Cu(CH_3_CN)_4_PF_6_, as an example. As depicted in Figure 2a, the reaction barrier of this process aligns with the optimal reaction temperature of 50 °C, further confirming that the intermediate state of the reaction is the *α*-imino copper carbene. 

To gain a better understanding of the reaction mechanism, we performed Density Functional Theory (DFT) calculations on three hypothetical pathways, as depicted in Figure 2b. In PATH 1A (represented by the blue line in Figure 2b), the interaction between diphenylphosphinic acid and copper carbene intermediate IM1 leads to the formation of intermediate IM1′. Subsequently, IM1′ proceeds to the copper-related intermediate IM2 via transition state TS2 with an activation energy barrier of only 3.3 kcal/mol. Once IM2 is formed, the copper catalyst dissociates through transition state TS3′ with a ∆G of 4.4 kcal/mol, resulting in the production of the free ylide IM3. This indicates a high likelihood of the copper catalyst departure. In TS3′, the β-C–Cu distance changes from 2.14 Å in IM2 to 2.53 Å, while the β-C–Cu distance in IM3 is 5.50 Å, indicating complete dissociation of the copper catalyst upon formation of IM3. Next, the proton migrates from diphenylphosphinic acid 2-2a’ to the β-C position of IM3 via transition state TS4, resulting in the formation of IM4 with an energy barrier of 38.8 kcal/mol. Subsequently, IM4 undergoes transformation into IM5 through intramolecular proton transfer, with an energy barrier of 47.6 kcal/mol, via TS5 from β-C to α-C. Finally, the proton is transferred from the α-C position of IM5 to the N atom of the Schiff base group, leading to the formation of the 3-phosphorylindole product (PD),with an energy barrier of 19.7 kcal/mol. 

Compared to PATH 1A, PATH 1B (indicated by the black line in Figure 2b) differs only in the simultaneous occurrence of proton transfer between diphenylphosphinic acid 2-2a’ and β-C and the dissociation of the copper catalyst after the formation of IM2 via transition state TS3. This results in the formation of IM4 with an energy barrier of 41.3 kcal/mol. PATH 1A and 1B explore the 1,1-insertion pathway of α-imino carbenes in P–H insertion reactions. However, the high activation energy barrier (47.6 kcal/mol) observed during the proton transfer process indicates that the 1,1-insertion pathway is unfavorable for the reaction. Additionally, this high activation energy barrier contradicts the reported reaction temperature of 50 °C in the literature. Thus, there must be another more reasonable reaction pathway for the P–H insertion reaction of α-imino carbenes. 

On the other hand, PATH 2 (represented by the red line in Figure 2b) corresponds to the 1,3-insertion pathway for the copper-catalyzed P–H insertion reaction of α-imino carbenes. The process of forming the copper-related intermediate IM3 in PATH 2 is similar to that in PATH 1A. Through transition state TS2, diphenylphosphinic acid 2-2a’ interacts with copper carbene intermediate IM1 to yield the copper-related intermediate IM2 (∆G = 3.3 kcal/mol). Once IM2 is formed, the copper catalyst dissociates through transition state TS3′ with a ∆G = 4.4 kcal/mol, producing the free ylide IM3. In IM3, there exists a strong hydrogen bond interaction (1.57 Å) between the hydroxyl group of the phosphinic acid and the oxygen atom of the sulfonyl group. Subsequently, in the presence of the Schiff base group, the hydroxyl group of the phosphinic acid forms a hydrogen bond (1.59 Å) with the nitrogen atom of the Schiff base group on the α-imino carbene through transition state TS5′ (5.2 kcal/mol). In the case of the existing Schiff base, the O–H group of phosphinous acid tended to form a hydrogen bond with the N atom of the Schiff base group in IM5′ via transition state TS5′ (5.2 kcal/mol). Consequently, the proton of the phosphinous acid could be captured by the Schiff base through transition state TS6′ (−1.1 kcal/mol) and finally generate 3-phosphonylindole (PD). This step played a key role in the 1,3-insertion pathway. This reaction pathway is deemed the most probable mechanism for the copper-catalyzed P–H insertion reaction of α-imino carbenes. 

**Figure 3:**
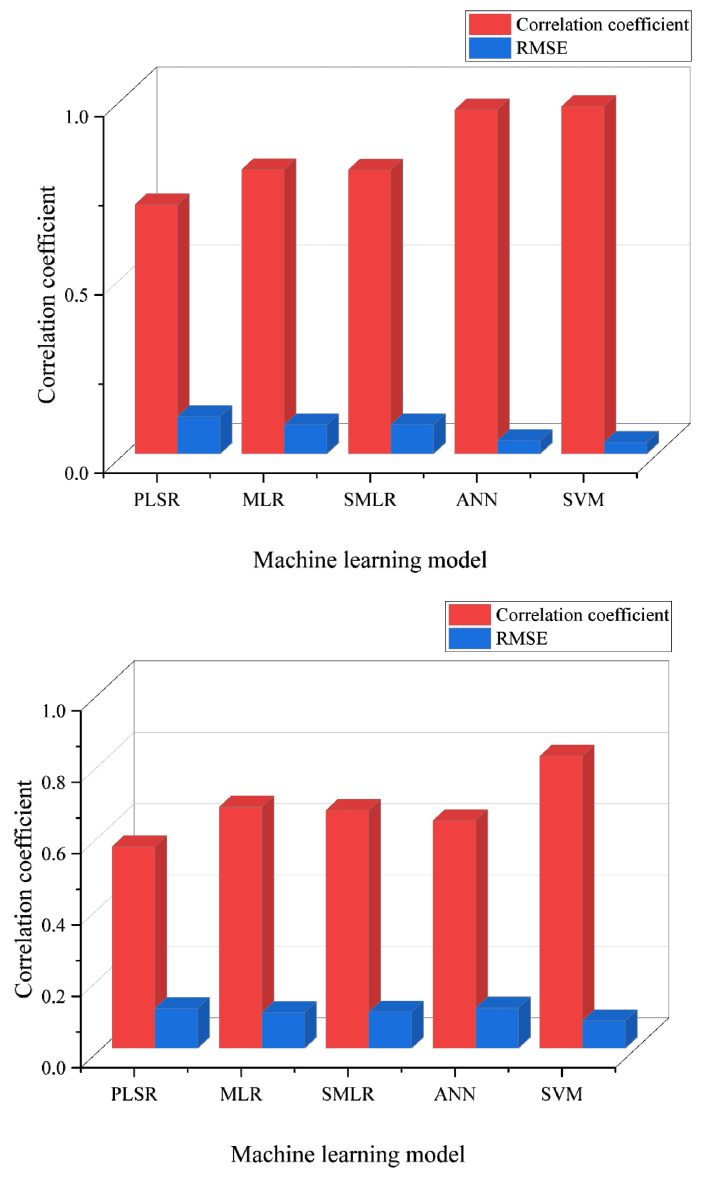
Regression coefficients and cross-validation results of various machine learning models.

Although the DFT model for transition states is accurate, it remains a challenging task to determine the yield of catalytic reactions solely based on this model. Consequently, we have decided to integrate the transition state model with AI methods to facilitate the determination of the transition state characteristics and the prediction of the reaction yield. Previous reports combining quantum chemical transition state models with machine learning analysis to predict the yield of copper-catalyzed P–H insertion reactions are scarce. One important clarification needs to be made: the descriptors we obtained were based on calculations performed using α-amino copper carbenes as the transition state, as the various indices in this step are crucial in determining the feasibility of the reaction. After identifying the reactive transition state, we extracted 16 atomic and molecular descriptors from the transition state model using quantum chemical calculations. It is worth noting that in order to obtain more accurate calculations and more persuasive results, the experimental impact brought by solvent effects was taken into consideration during the establishment of the transition state model. However, in the subsequent machine learning modeling process, it was found that the solvent effects had little influence on the final prediction results. Moreover, considering the computational cost and time consumption in quantifying parameters, it was ultimately decided not to further consider solvent effects. These descriptors, along with the reaction yield, were utilized as the input and output datasets for our machine learning model. These descriptors, which may potentially impact the experimental catalytic yield, encompass the catalyst and reaction molecular mass (Mass), lipophilicity (Log P), water solubility (Log S), transition state energy (E-RB3LYP), dipole moment, polarizability, heat of formation (E (Thermal)), heat capacity, entropy, lowest occupied molecular orbital (LUMO), highest occupied molecular orbital (HOMO), length of Cu–C bond (Length (Cu–C)), length of P–C bond (Length (P–C)), Mulliken charge of Cu (Mulliken (Cu)), Mulliken charge of P (Mulliken (P)), and Mulliken charge of C (Mulliken (C)). Detailed calculation results for all data descriptors can be found in SI 1. To predict the performance and transition state characteristics of Cu catalysts, we employed five machine learning models: Partial Least Squares Regression (PLSR); Multiple Linear Regression (MLR); Stepwise Multiple Linear Regression (SMLR); Artificial Neural Networks (ANN); and Support Vector Machine Regression (SVM). Each machine learning model provides prediction results based on its inherent algorithm, and comprehending the differences among them is crucial for selecting the most appropriate model for practical applications. In the field of computational chemistry and cheminformatics, the SVM is widely used for identifying new active compounds. Additionally, Support Vector Regression (SVR) has emerged as the preferred method for modeling non-linear structure–activity relationships and predicting compound potencies [42,43,44,45,46]. In our study, the SVM demonstrated the highest prediction accuracy among the five tested machine learning models, as well as the best performance in cross-validation (Figure 3). Therefore, we opted to utilize the SVM for further analysis. 

**Figure 4:**
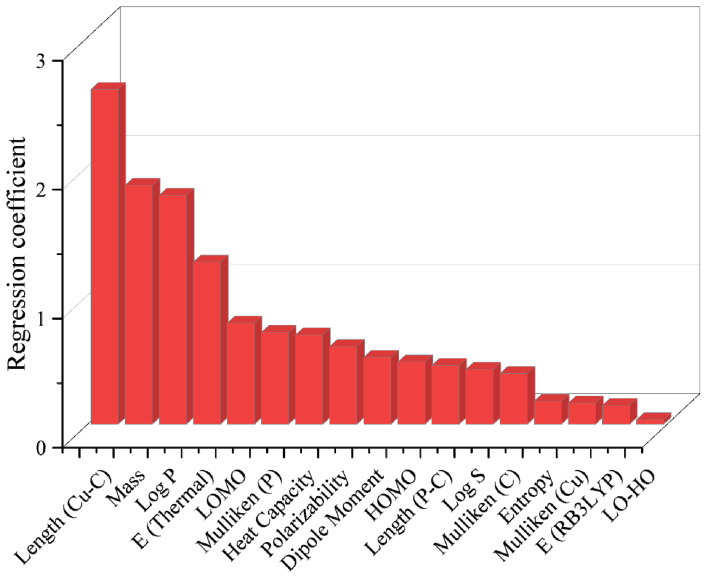
Importance plots of 16 descriptors.

After selecting the Support Vector Machine (SVM) as the optimal model, we further investigated the critical features that significantly affect the catalytic efficiency using the Principal Component Analysis (PCA) method, as illustrated in Figure 4. PCA is a multivariate statistical technique [47] that examines the correlations among multiple variables and explores how to reveal the internal structure of these variables by deriving a few principal components. These components preserve as much information as possible from the original variables while being uncorrelated. The identified descriptors, including length (Cu-C), Mass, Log P, and E (Thermal), align with expert chemists’ intuitions regarding the relative importance of catalyzing P–H insertion reactions. The graph in Figure 4 clearly demonstrates that these descriptors have regression coefficients exceeding 1. Traditional analytical methods face challenges in quantifying these parameters accurately, but the combination of quantum chemistry calculations and machine learning provides a straightforward and direct approach. 

**Figure 5:**
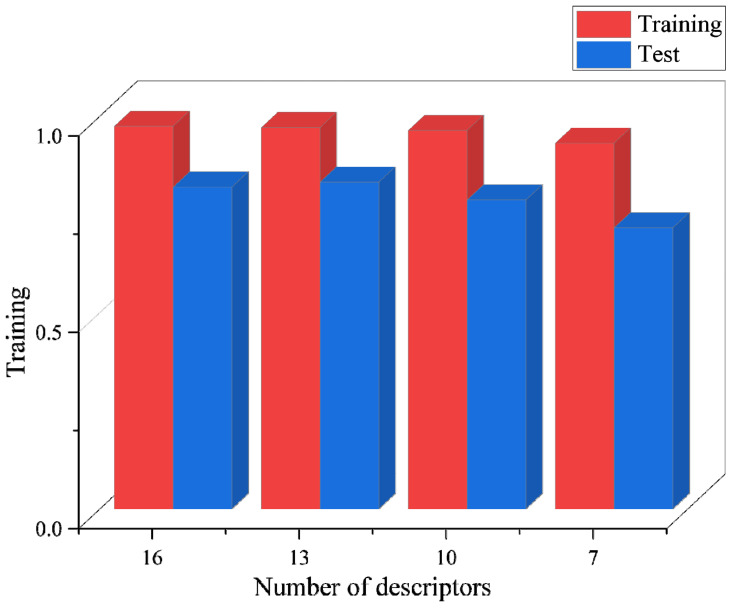
Model accuracy for different numbers of descriptors.

To investigate the effect of the number of descriptors on predictive performance, we trained SVM models based on the top 16, 13, 10, and 7 descriptors shown in Figure 5. This demonstrated that reducing the number of descriptors from 16 to 13 has a negligible impact on predictive accuracy, and it further suggests that even when considering only the top 13 descriptors, the SVM model can provide satisfactory predictive results. Moreover, this indicates that the initially considered and selected descriptors are representative and accurately capture the factors influencing catalytic reaction outcomes. 

The descriptors were obtained from quantitative calculations, which encompass a wide range of categories necessary for predicting the yield of the P–H insertion reaction. However, this also increases the complexity and computational time of the calculations. Additionally, due to the “black-box” nature of machine learning algorithms, we aim to establish a connection between this work and practical applications by providing interpretability rather than just predictive capabilities. Therefore, we conducted an importance analysis of these descriptors, gradually reducing their number and sequentially modeling them to observe their impact on prediction accuracy in order to gain a deeper understanding of these descriptors.

It can be observed that although the top four important descriptors contribute significantly to the overall importance, the prediction accuracy of the model gradually decreases as the number of descriptors is reduced. Even when only the top seven descriptors are used, the model can still achieve a cross-validation regression coefficient close to 0.8. However, this does not imply that descriptors other than the top-ranked ones are unimportant; their combination provides higher predictive accuracy. Our work offers a choice for situations where extensive calculations are not feasible, but an approximate estimation of reaction yield is needed. In such cases, only the top few important descriptors can be selected for modeling.

**Figure 6:**
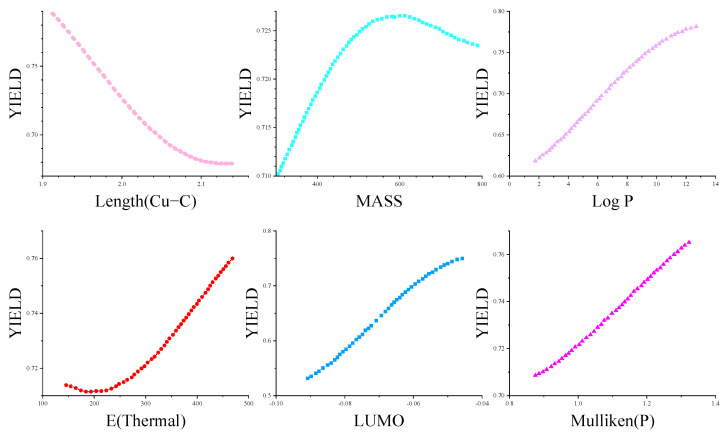
Sensitivity graph of each descriptor.

Further examination of Figure 6 reveals that length (Cu–C), Mass, Log P, E (Thermal), LOMO, and Mulliken (P) are the most influential factors affecting catalytic yield. To gain additional chemical insights, we obtained sensitivity plots for these descriptors from the SVM model, as shown in Figure 6. These plots display the catalytic yield as a function of descriptor variation. The results indicate that the reaction yield increases with the increase in Log P, E (Thermal), LOMO, and Mulliken (P) descriptors, while it decreases with the increase in length (Cu–C) of the molecule. The highest yield is observed at a molecular weight of approximately 500, which aligns with the expected trends and insights of chemists. The purpose of conducting sensitivity analyses is to establish a connection with real-world experiments, enhancing interpretability and providing guidance for the further exploration of reactions. For instance, in the P–H insertion reaction, an increase in Cu–C distance implies a decrease in the likelihood of the reaction occurrence. The molecular weight of the transition state should fall within an appropriate range to facilitate the reaction, as deviations towards larger or smaller values can lead to a decrease in yield. Given that most reactions occur in organic solvents, a stronger lipophilicity is associated with higher yields. Hence, it is important to consider the lipophilicity of reactants in subsequent experimental processes. However, despite the intuitive nature of variables such as molecular weight and lipophilicity, other descriptors, though informative in understanding their significance and impact on yield variations, are not easily controlled, posing limitations in experimental design. 

**Figure 7:**
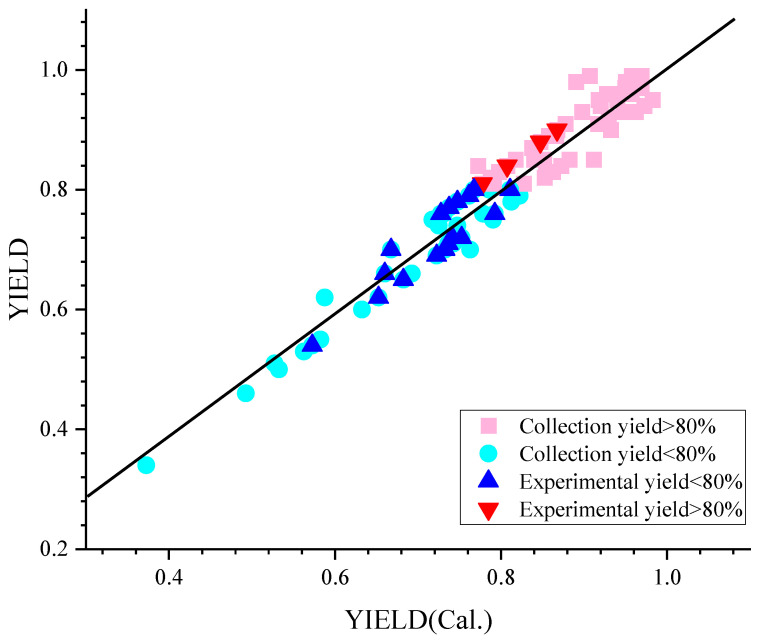
Graph of experimental results and predicted curve.

To demonstrate the reliability of the constructed model in a more intuitive manner, we conducted experiments on 26 synthetic design schemes, and the final experimental results and model prediction curves are shown in SI 2 and Figure 7. Among these schemes, the pink squares and blue circles represent samples with yields >80% and ≤80%, respectively, collected from other literature sources. The red inverted triangles and blue triangles represent 26 samples with yields >80% and ≤80%, respectively, obtained in this experiment. Although slight discrepancies exist between the experimental and calculated values, mainly due to differences in experimental conditions and idealization of the simulation model, Figure 7 demonstrates that the accuracy and reliability of our established prediction model can be further verified through experiments. 

## 3. Experimental Section 

### 3.1. Data Source

All the data used for modeling in this paper were sourced from five publicly available literature publications that discuss copper-catalyzed P–H insertion reactions [48,49,50,51,52]. 

### 3.2. Quantum Chemistry Calculations and Descriptor Acquisition

All theoretical calculations in this chapter were conducted using the Gaussian 16 software package [53] based on density functional theory (DFT). The B3LYP-D3 density functional [54,55,56,57,58] was employed for geometry optimization of all reaction stationary points. The metal copper atoms were modeled using the LANL2DZ [59,60] pseudo-potential basis set, while the 6-31G(d) basis set [61] was used for all other atoms. Frequency calculations were performed to determine whether the stationary points corresponded to minimum values or first-order saddle points. In addition, intrinsic reaction coordinate (IRC) calculations were carried out at the same theoretical level [62,63,64] to confirm the connectivity between the relevant reactants and products, thereby verifying the accuracy of the transition state. 

For all single-point energy calculations, the optimized structures obtained from the geometry optimization were used as a basis at the B3LYP-D3/6-31G(d)-LANL2DZ level. Furthermore, the 6-311++G(d,p) basis set [65,66] was utilized for all atoms except copper, and the M06 algorithm [67] was employed for these calculations. To account for solvent effects, the Truhlar and Cramer-developed SMD solvent model [68] was employed. The solvent correction was performed using the SMDCHCl3/M06/6-311++G(d,p)-LANL2DZ theoretical level. The Gibbs free energy was calculated at a temperature of 323.15 K and a pressure of 1 atm, based on the actual reaction temperature of 50 °C. 

### 3.3. Machine Learning Models

The model construction and testing in this study were carried out using analytical software (ExMiner 1.8.7.8) developed by our laboratory [69]. The ML predictions rely on the selection of algorithms, and even experienced data scientists cannot determine the best-performing algorithm without experimenting with different ones. Hence, in this study, five ML models were established utilizing preprocessed datasets. 

#### 3.3.1. Partial Least Squares Regression (PLSR)

The principle behind PLSR [70] is to find a linear regression model by projecting the predictor variables and the observed variables onto a new space through.

#### 3.3.2. Multiple Linear Regression (MLR)

The basic principle of MLR [71] is similar to that of simple linear regression, with the difference being the involvement of two or more independent variables.

#### 3.3.3. Stepwise Multiple Linear Regression (SMLR)

SMLR [72] analysis involves the gradual introduction of variables, with each new variable being tested against the previously selected variables to ensure that each variable in the resulting subset is significant. The process is repeated until no further variables can be added.

#### 3.3.4. Artificial Neural Networks (ANN)

ANN [73] is a computational model composed of a large number of interconnected nodes (or neurons). Each node represents a specific output function, known as an activation function. The connection between any two nodes represents a weighted value for the signal passing through that connection, known as a weight. Artificial Neural Networks simulate human memory through this mechanism. The output of the network depends on its structure, connection pattern, weights, and activation functions.

#### 3.3.5. Support Vector Machine (SVM)

The Support Vector Machine (SVM) is a powerful machine learning algorithm that is widely used in classification and regression tasks. The core concept of the SVM is to find a hyperplane that maximizes the margin between two classes of data, allowing for effective classification. The choice of kernel function is crucial in the SVM, as it determines how the data are transformed and classified [74]. 

In regression tasks, Support Vector Machine Regression (SVR) is a significant application of the SVM. SVR aims to find a regression plane that minimizes the distance between all data points and the plane. This approach allows for the accurate prediction and modeling of continuous variables. 

To apply the SVM to regression, an alternative loss function is introduced. The results obtained via SVR have shown promising performance. The key idea behind SVR is to map the input data X into a higher-dimensional feature space F using a non-linear mapping function Φ. Regression is then performed in this transformed space, enabling the modeling of complex relationships between variables. 

In practical applications, non-linear models are often necessary for better data fitting. Similar to the non-linear support vector classification approach, non-linear mapping can be employed to transform the data into a higher-dimensional feature space. In this transformed space, linear regression can be applied to accurately model the data. 

The complete SVM algorithm can be described in terms of dot products between data points. The dot product measures the similarity between two data points and is used to determine the position of the hyperplane that separates the different classes. By maximizing the margin between classes, the SVM ensures robust classification and regression results. 

In conclusion, the Support Vector Machine is a versatile algorithm that can be used for both classification and regression tasks. SVM regression (or SVR) allows for the accurate prediction of continuous variables by finding a regression plane that minimizes the distance to the data points. Non-linear models can be achieved through the use of kernel functions and by mapping the data into a higher-dimensional feature space. The dot product between data points plays a crucial role in determining the position of the hyperplane and achieving optimal classification and regression results. 

### 3.4. Synthesis

**Scheme 1:**
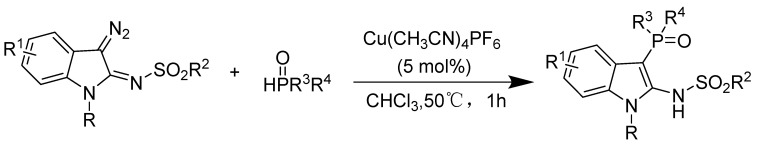
Overall design scheme of the P–H insertion reaction.

**Scheme 2:**
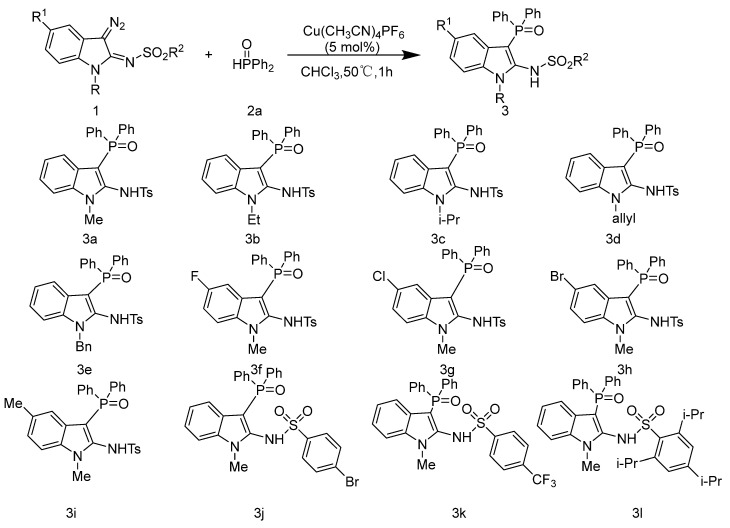
Partial design scheme of the P–H insertion reaction.

**Scheme 3:**
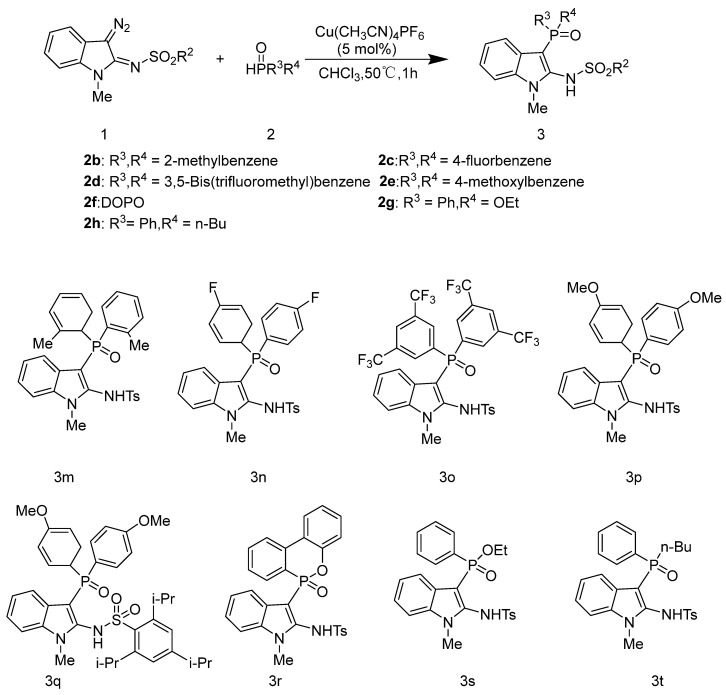
Partial design scheme of the P–H insertion reaction.

**Scheme 4:**
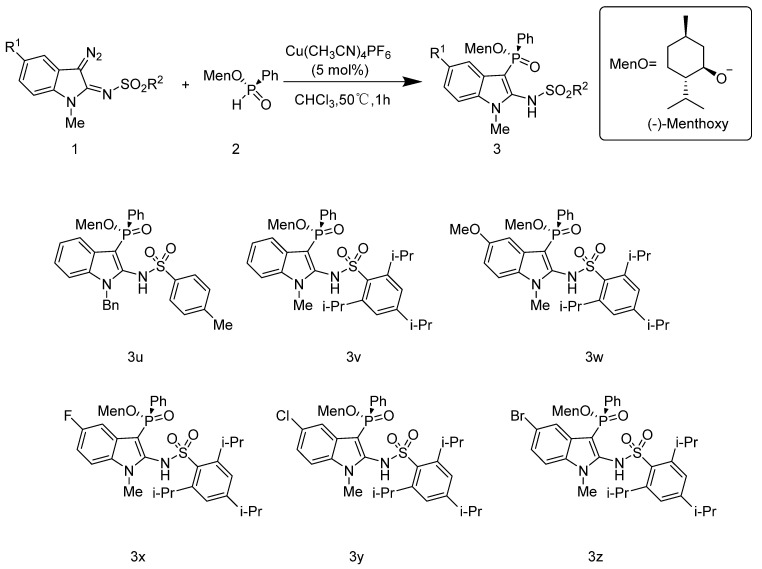
Partial design scheme of the P–H insertion reaction.

Based on previous research conducted in our laboratory, a reaction scheme for copper-catalyzed P–H insertion was devised, as depicted in Scheme 1, Scheme 2, Scheme 3 and Scheme 4. The design and discussion encompassed the substitution of both the 1st and 5th positions of the indole substrate, along with various sulfonyl groups. Moreover, the different types of H-type phosphine oxides and the subsequent modifications in experimental outcomes resulting from the addition of chiral reagents were taken into consideration. To begin the experiment, a mixture of 3-azidoindole-2-imine (0.2 mmol) and 0.4 mL of CHCl_3_ was gradually added to a mixed solution containing H-type phosphine oxide (0.2 mol), catalyst Cu(CH_3_CN)_4_PF_6_ (0.01 mmol), and 0.4 mL of CHCl_3_. The reaction mixture was then stirred at 50 °C under an argon atmosphere for one hour. Upon completion, the solvent was evaporated under vacuum, and the resulting residue was purified using silica gel column chromatography (petroleum ether/ethyl acetate 3:1) to obtain the phosphine hydride compound. 

### 3.5. Structural Validation of Synthesized Product

The ^1^H NMR and ^13^C NMR spectra were acquired using a Brucker 600 MHz spectrometer in CDCl_3_. TMS served as the internal standard for ^1^H NMR (δ = 0), while CDCl_3_ was employed as the internal standard for ^13^C NMR (δ = 77.0). Additionally, the ^31^P NMR and ^19^F NMR spectra were recorded on the same instrument. Chemical shifts were reported in parts per million (ppm), and the multiplicity was indicated as s (singlet), d (doublet), t (triplet), q (quartet), m (multiplet), or br (broad). High-resolution mass spectrometry (HRMS) using electrospray ionization (ESI) was performed on a Thermo Fisher Scientific LTQ FT Ultra. The starting materials were purchased from Aldrich, Macklin, and Energy Chemicals and were used without further purification. Solvents were dried and purified following the procedures. Column chromatography was conducted using silica gel (200–300 mesh ASTM). The substrates were prepared according to published procedures [75]. 

## 4. Conclusions

This work presents a novel approach that combines quantum chemical transition state modeling with machine learning to establish a highly accurate model for predicting transition state features and yield in P–H insertion reactions. This study proves the potential of integrating quantum mechanical calculations and machine learning techniques to predict the outcome of catalytic reactions, which could significantly reduce the costs of human labor and experimentation. Furthermore, by developing appropriate descriptors and fine-tuning hyperparameters, this method can be extended to other organic and inorganic material fields, thereby facilitating the improvement and discovery of new materials. Furthermore, it is important to recognize that there is still room for further improvement in our work—for example, exploring the inclusion of solvent-related descriptors to enhance the general applicability and stability of the prediction model. Additionally, conducting sensitivity analyses on different descriptors would provide theoretical guidance for subsequent experimental designs, rather than relying solely on designing predictions before conducting experiments. This approach would enhance the practicality and functionality of the model, and it is an area we will explore in our future investigations.

## Data Availability

All relevant data generated during our research process have been uploaded to the Supplementary Files.

## Supplementary Materials

The following supporting information can be downloaded at: https://www.mdpi.com/article/10.3390/molecules28165995/s1
